# Pilomatrix-like High-Grade Endometrioid Carcinoma of the Ovary: Case Report, Literature Review, and Differential Diagnosis

**DOI:** 10.3390/diagnostics12123146

**Published:** 2022-12-13

**Authors:** Angela Santoro, Antonio Travaglino, Michele Valente, Damiano Arciuolo, Giulia Scaglione, Nicoletta D’Alessandris, Stefania Sfregola, Francesca Addante, Caterina Fulgione, Antonio Raffone, Angelo Minucci, Frediano Inzani, Gian Franco Zannoni

**Affiliations:** 1 Pathology Unit, Department of Woman and Child’s Health and Public Health Sciences, Fondazione Policlinico Universitario Agostino Gemelli IRCCS, 00168 Rome, Italy; 2 Pathology Unit, Department of Advanced Biomedical Sciences, School of Medicine, University of Naples Federico II, 80131 Naples, Italy; 3 Pathology Institute, Catholic University of Sacred Heart, 00168 Rome, Italy; 4 Department of Emergency and Organ Transplantation, Section of Anatomic Pathology, School of Medicine, University “Aldo Moro”, 70121 Bari, Italy; 5 Gynecology and Obstetrics Unit, Department of Neuroscience, Reproductive Sciences and Dentistry, Federico II University of Naples, 80131 Naples, Italy; 6 Division of Gynecology and Human Reproduction Physiopathology, Department of Medical and Surgical Sciences (DIMEC), IRCCS Azienda Ospedaliero-Univeristaria di Bologna, S. Orsola Hospital, University of Bologna, 40138 Bologna, Italy; 7 Molecular and Genomic Diagnostics Unit, Fondazione Policlinico Universitario Agostino Gemelli IRCCS, 00168 Rome, Italy

**Keywords:** endometrioid carcinoma, ovarian cancer, pilomatrix carcinoma, ghost cell, shadow cell, squamous, basaloid, histotype, keratinization

## Abstract

Pilomatrix-like high-grade endometrioid carcinoma (PiMHEC) has recently been described as an aggressive variant of endometrial carcinoma. Herein, we described a case of ovarian PiMHEC, comparing it to endometrial PiMHEC and assessing previously published cases of putative ovarian PiMHEC. A 65-year-old woman underwent hysterectomy for an ovarian tumor characterized by solid nests of basaloid cells with prominent ghost cell keratinization. Immunohistochemistry showed nuclear β-catenin and CDX2 expression and loss of estrogen and progesterone receptors and PAX8. These features were consistently observed in all previously published cases and may represent diagnostic criteria of PiMHEC. Other frequent features were geographic necrosis and a low-grade endometrioid component. CK7, neuroendocrine, and basal/squamous markers were inconsistently expressed. All cases with available follow-up showed poor prognosis. PiMHEC should be distinguished from mimickers, such as high-grade endometrioid carcinoma with geographic necrosis, low-grade endometrioid carcinoma with ghost cell keratinization, and undifferentiated/dedifferentiated carcinoma. In conclusion, PiMHEC can also occur in the ovary and shows several consistent clinical, morphological, and immunophenotypical features. These features support that PiMHEC is a distinct entity requiring an aggressive management.

## 1. Introduction

Ovarian carcinoma is a heterogeneous group of epithelial malignant neoplasms of the ovary [1,2]. Despite being less common than endometrial carcinoma, ovarian carcinoma represents the most lethal malignant tumor of the female genital tract, being included among the five leading causes of cancer deaths in women [3]. The different types of ovarian carcinoma show crucial differences in terms of etiopathogenesis, histological features, molecular background, and biological behavior. More than 95% of all cases of ovarian carcinoma belong to one of the five major histotypes: high-grade serous carcinoma, which accounts for about 70% of ovarian carcinomas and mostly arise in the fallopian tube epithelium; endometrioid carcinoma and clear cell carcinoma, which account for about 10% of cases each and arise from ectopic endometrial epithelium (i.e., endometriosis); mucinous carcinoma, which accounts for less than 5% of cases and is of undefined origin (can be associated with Brenner tumor and with teratoma); low-grade serous carcinoma, which accounts for less than 5% of cases and arises in tubal-type epithelium [1,2].

Ovarian endometrioid carcinoma is morphologically and biologically analogous to its endometrial counterpart. Compared to the latter, ovarian endometrioid carcinoma shows a higher frequency of *CTNNB1* mutations and a lower frequency of *POLE* mutations. Recent studies have shown that ovarian endometrioid carcinoma may be stratified according to the four molecular prognostics of endometrial carcinoma, developed based on the findings by The Cancer Genome Atlas (TCGA) Research Network. Four prognostic groups are: *POLE*-mutated (or “ultramutated” group), associated with very high mutational load and favorable prognosis; microsatellite instability/mismatch repair-deficient (or “hypermutated” group), associated with high mutational load and intermediate prognosis; p53-abnormal (or “ copy number-high” group), associated with high copy number alterations and poor prognosis; “no specific molecular profile” (or “copy number-low” group), characterized by the absence of the molecular signatures of the other groups and intermediate prognosis. These groups may have the potential to improve the prognostic stratification and management of ovarian endometrioid carcinoma [4]. 

Endometrioid carcinoma can show several different types of altered differentiation and unusual growth patterns, which may mimic other tumors and constitute diagnostic pitfalls. The most common types of altered differentiation found in endometrioid carcinoma are squamous differentiation, morular metaplasia, and mucinous differentiation; tubal (ciliated) differentiation and cytoplasm eosinophilia or clearing may also occur. Cell spindling, papillary growth, transitional cell morphology, and sertoliform (sex cord-like) pattern are among the diagnostically challenging morphologic variants [5]. In addition to altered differentiation and growth pattern, endometrioid carcinoma can also show evolution into more aggressive histotypes. For instance, neuroendocrine and undifferentiated carcinomas of the female genital tract may derive from transformation of a preexisting endometrioid carcinoma [6,7].

Pilomatrix-like high-grade endometrioid carcinoma (PiMHEC) is a recently described variant of endometrial carcinoma. PiMHEC is characterized by a high-grade solid basaloid appearance with extensive geographic necrosis and diffuse ghost cell keratinization; these features are reminiscent of pilomatrix carcinoma, a malignant cutaneous adnexal neoplasm exhibiting hair matrix differentiation. PiMHEC has been considered as a variant of endometrioid carcinoma based on the association with a low-grade endometrioid component. Immunohistochemically, PiMHEC typically shows a diffuse nuclear β-catenin expression, which reflects underlying *CTNNB1* exon 3 mutations. Regarding prognosis, PiMHEC is associated with exceedingly poor oncologic outcomes [8,9]. The aggressive behavior of PiMHEC underlines the importance of recognizing this entity in the common practice. To the best of our knowledge, PiMHEC has not been described in the ovary. However, there are previously published reports of ovarian tumors resembling pilomatrix neoplasms [10,11,12]; it cannot be excluded that these tumors represented cases of ovarian PiMHEC.

In this study, we report a clinicopathological and immunohistochemical analysis of a case of ovarian PiMHEC. Moreover, we reviewed all the previously published cases of ovarian carcinomas showing features that could be consistent with PiMHEC. We focused on the diagnostic criteria and differential diagnosis of PiMHEC, in order to favor the identification of this aggressive entity in the common practice.

## 2. Case Presentation

**Figure 1:**
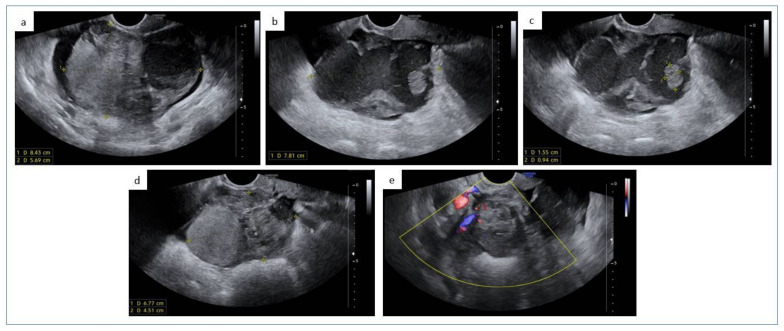
(**a**–**e**) In right adnexal site a multilocular solid-cystic mass (88 × 57 × 78 mm) has been observed, with ground grass content, complete/incomplete septa (**a**,**b**) and endoluminal papillae (15 × 9 mm, the largest one, (**c**)). The solid component measured 68 × 45 mm (**d**) and it was moderately vascularized at Color Doppler (**e**).

The patient was a 65-year-old woman with no family history of cancer. She had sudden onset of abdominal pain and visited at our hospital (emergency department). A high echoic right ovarian multilocular mass (83 × 57 × 78 mm), with ground glass content and few endoluminal papillae, was shown by ultrasound examination, with moderate vascularization at color Doppler (Figure 1).

**Figure 2:**
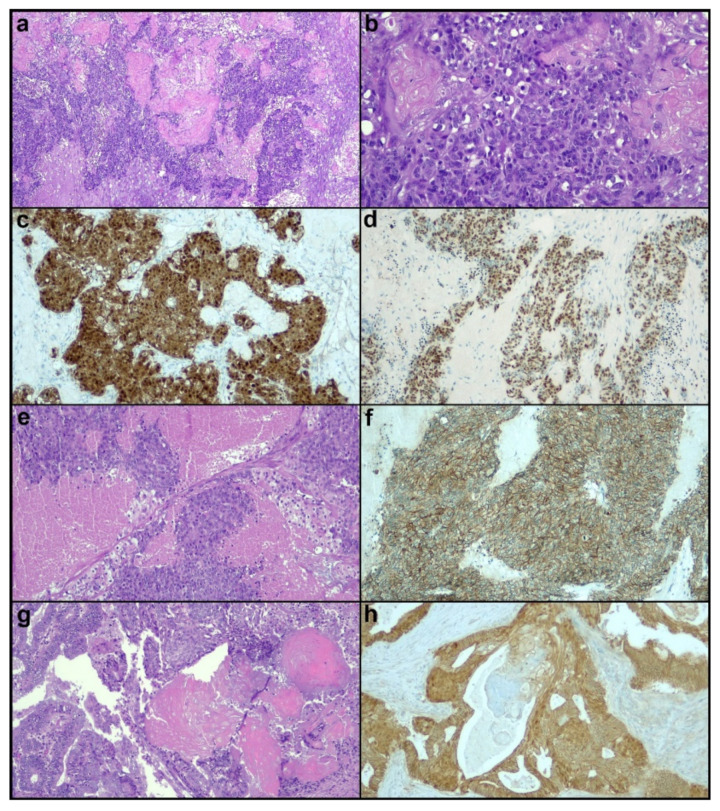
(**a**–**d**) Ovarian PiMHEC characterized by solid nests of basaloid cells with prominent ghost cell keratinization ((**a**,**b**), hematoxylin-eosin, magnification 40× and 200×), nuclear β-catenin expression ((**c**), 100×) and CDX2 positivity ((**d**), 100×). (**e**,**f**) High-grade endometrioid carcinoma with basaloid cells and prominent necrosis; note the absence of ghost cells ((**e**), hematoxylin-eosin, 100×) and the membrane β-catenin expression ((**f**), 100×). (**g**,**h**) Low-grade endometrioid carcinoma with morular metaplasia and ghost cells; there is an admixture of morular metaplasia and low-grade glandular structures, with no high-grade basaloid cells ((**g**), hematoxylin-eosin, 100×); β-catenin immunostaining highlights the presence of morular metaplasia (nuclear expression) and glandular structures (membrane expression) ((**h**), 100×).

Increased CA 125 values were documented (127 U/mL). Intraoperative frozen sections showed a carcinoma with a solid growth pattern. The patient underwent hysterectomy with bilateral salpingo-oophorectomy, omentectomy and peritoneal biopsies by laparoscopy; the ovarian mass was removed by in bag morcellation. Histological examination revealed a solid carcinoma characterized by high-grade basaloid cells with areas of necrosis and diffuse ghost cell keratinization (Figure 2a,b), which extended beyond the ovary and infiltrated the fallopian tube. The carcinoma showed a minor low-grade endometrioid component and appeared to arise in a borderline seromucinous tumors. Several keratin granulomas were observed in the surrounding tissues. Immunohistochemically, the tumor showed diffuse nuclear expression of β-catenin and CDX2 (Figure 2c,d). Neuroendocrine markers chromogranin and synaptophysin were focally expressed, while Müllerian markers PAX8, estrogen receptor, and progesterone receptor were completely negative. The tumor was positive for cytokeratin 7, while p63 was focally expressed. Mismatch repair proteins expression were preserved, and p53 expression was wild-type. Molecular analysis showed mutations in *CCDN1*, *CTNNB1*, *ARID1A*, and *PIK3CA*. The described features were widely overlapping with those described for PiMHEC of the endometrium. On this account, we interpreted the tumor as an ovarian PiMHEC. Final tumor stage was FIGO IIA. After thorough counseling, the patient decided to avoid lymph node resection. The patient is currently being treated with carboplatin + taxol.

## 3. Literature Review

We performed a review of the literature to identify previously published cases of ovarian neoplasms which showed morphological and immunophenotypical features consistent with PiMHEC. We used PubMed, Scopus, and Web of Science as electronic databases. The electronic search was performed from the inception of each database to August 2022, based on a combination of the following text words: “ovarian”, “ovary”, “carcinoma”, “endometrioid”, “squamous”, “basaloid”, “keratinizing”, “keratinization”, “ghost cells”, “shadow cells”, “β-catenin”. Article titles were screened first to identify potentially suitable articles. A second screening step was based on the abstracts. Only articles that were considered suitable based on the abstract were full-text screened. Eligible studies were included if they described an ovarian neoplasm constituted of a solid basaloid component with ghost cell keratinization. At the end of the research process, we identified three previously published cases of ovarian tumors which appeared consistent with PiMHEC (Table 1).

The first study was published in 1996 by Fang and colleagues [10]. The patient was a 48-year-old woman with a 13 cm ovarian mass. The tumor was confined to one ovary and was associated with endometriosis. The authors histologically described the tumor as a “pilomatricoma-like endometrioid adenosquamous carcinoma with neuroendocrine differentiation”; to date, the term “adenosquamous carcinoma” is no more used in ovarian and endometrial neoplasms. Such a definition was used for carcinomas composed of a glandular component and a squamous component showing overtly malignant features. By contrast, “adenoacanthoma”, indicated cases in which the squamous component showed a benign morphology. Both “adenosquamous carcinoma” and “adenoacanthoma” of the ovary and endometrium tumors are now regarded as endometrioid carcinomas with squamous differentiation [13]. Although the expression of β-catenin, CDX2, and Müllerian markers was not assessed, we considered this tumor to be a putative PiMHEC based on the high-grade solid features, the extensive coagulative tumor cell necrosis, and the diffuse ghost cell keratinization. In addition, the tumor showed patchy positivity for neuroendocrine markers, as has been described in PiMHEC of the endometrium. The only concern we had with this case is the reported presence of a glandular component. The authors stated the tumor was of high-grade and did not describe a low-grade component. It is therefore difficult to define the nature of such a component. Unfortunately, there were no available follow-up data.

The second study was published in 2010 by Lalich and colleagues [11] and described a 31-year-old woman with a 9 cm ovarian mass. The tumor was described as an “ovarian carcinoma with shadow cells mimicking a pilomatrical neoplasm”. Consistently with PiMHEC, the tumor showed a high-grade solid basaloid component with extensive coagulative tumor cell necrosis and diffuse ghost cell keratinization; a minor low-grade endometrioid component was also observed. The tumor was diffusely positive for β-catenin (nuclear staining) and negative for cytokeratin 7, estrogen, and progesterone receptors. Neuroendocrine markers were negative, as well as basal/squamous cell markers. The neoplasm showed highly aggressive behavior, with abdominopelvic carcinomatosis, visceral and cutaneous metastases. The patient died of disease at six months. Based on these features, we considered this case as a bona fide ovarian PiMHEC.

The third study was published in 2014 by Zamecnik and colleagues [12], describing a 45-year-old woman with a 14 cm ovarian mass. The tumor was deemed “ovarian basaloid carcinoma with shadow cell differentiation” and showed morphological and immunophenotypical features analogous to the case described by Lalich et al., with diffuse nuclear staining for β-catenin, negativity for cytokeratin 7, estrogen and progesterone receptors, and neuroendocrine markers. The only remarkable differences were the absence of an evident low-grade endometrioid component and the positivity for basal/squamous cell markers (i.e., p63, cytokeratin-5/6, and cytokeratin-34βE12). This tumor showed an aggressive behavior with nodal and visceral metastases, which led the patient to death by disease at eight weeks of follow-up. Further, in this case, we were confident that the described tumor represented a bona fide ovarian PiMHEC.

Clinicopathological features of the previously described cases of putative ovarian PiMHEC are reported in Table 1, while immunohistochemical features are reported in Table 2.

## 4. Discussion

This study reported a clinicopathological and immunohistochemical analysis of a PiMHEC of the ovary. A detailed review of the previously published cases of putative ovarian PiMHEC was also provided.

PiMHEC has been systematically described only recently. In 2021, Weisman et al. reported a series of five cases of endometrial carcinoma showing resemblance to pilomatrix carcinoma. The distinctive histological features were the solid growth with high-grade basaloid cells, the prominent geographic necrosis, and the ghost cell keratinization. The authors highlighted that all cases were associated with a low-grade endometrioid component, which support the derivation of PiMHEC from conventional endometrioid carcinoma. The authors also noted that all cases showed diffuse nuclear expression of β-catenin, which was found to be associated with mutations in the exon 3 of *CTNNB1* (β -catenin-encoding gene). On this basis, the authors labeled this tumor as “FIGO grade 3 endometrioid adenocarcinomas with diffusely aberrant β-catenin expression”. The nuclear expression of β-catenin was accompanied by positivity for CDX2, as it has been described in morular metaplasia of the endometrium [13]. The prognosis of tumors in the series by Weisman et al. was exceedingly poor, with all cases showing advanced stage, and with three in five patients being dead of disease within 14 months. The remaining two patients were either lost to follow-up or too recent to assess the survival time. Additional features reported by Weisman et al. were negativity for PAX8 and estrogen receptor, variable expression of neuroendocrine markers (chromogranin 2–10% of tumor cells; synaptophysin 20–60% of tumor cells), patchy p16 expression, wild-type p53 expression, retained mismatch repair protein expression; basal/squamous marker p63 was expressed in a minority of tumor cells in two cases. Based on the lack of aberrant p53 expression, the lack of mismatch repair protein loss of expression, and the aggressive behavior inconsistent with the presence of *POLE* exonuclease domain mutations, the authors suggested that these tumors are typically of “no specific molecular profile” according to the TCGA-based molecular classification [8].

Our research group subsequently described an additional case of endometrial carcinoma showing similar features to the cases described by Weisman et al. We suggested that the consistent morphological and immunophenotypical features of these tumors might warrant their recognition as a distinct entity. We adopted the term “PiMHEC” to refer to these neoplasms. The only difference in our case was the presence of a deficient mismatch repair protein expression, which demonstrated that not all PiMHECs are of “no specific molecular profile” [9].

In the current study, the described case highlights that PiMHEC can also occur in the ovary. We reviewed the previous literature for case of ovarian tumor showing resemblance with pilomatrix neoplasms and identified three cases of putative ovarian PiMHEC [10,11,12]. Our case and the previously published cases of ovarian PiMHEC showed several crucial analogies with the reported cases of endometrial PiMHEC. The distinctive morphology with solid growth, basaloid cells, necrosis, and ghost cell keratinization were consistently observed in the four cases of ovarian PiMHEC. A low-grade endometrioid component was only observed in two cases, including our one; another case was associated with ovarian endometriosis, which is a precursor of ovarian endometrioid carcinoma and supports the endometrioid lineage of PiMHEC [2,14]. Immunohistochemically, all cases tested for β-catenin showed the distinctive diffuse nuclear expression of the protein, suggesting the presence of underlying *CTNNB1* exon 3 mutations; CDX2 was also positive in these cases. In spite of the basaloid and keratinizing features of PiMHEC, basal/squamous markers (such as p63, cytokeratin-5/6, cytokeratin-34βE12) were not consistently expressed, as previously shown by Weisman et al. in endometrial PiMHEC. Cytokeratin 7 was positive in only one of three ovarian PiMHEC, in contrast with Weisman’s series which showed consistent positivity. Since the high-grade solid basaloid component of PiMHEC may resemble a large cell neuroendocrine carcinoma component, all reported cases of endometrial and ovarian PiMHEC were tested for neuroendocrine markers. While all cases of endometrial PiMHEC showed at least focal expression of neuroendocrine markers, two out of four cases of ovarian PiMHEC were completely negative. Remarkably, we recently observed another case of endometrial tumors with overt PiMHEC features which was negative for neuroendocrine markers. An interesting feature of both endometrial and ovarian PiMHEC is the consistent loss of Müllerian markers, such as PAX8 and estrogen and progesterone receptors. This finding suggests a loss of Müllerian differentiation and strengthens the idea that PiMHEC should be regarded as a distinct entity. In our case, molecular analysis revealed mutations in *CTNNB1* (which is consistent with nuclear β-catenin expression), *CCDN1*, *ARID1A,* and *PIK3CA* (which are consistent with the endometrioid lineage of PiMHEC).

Regarding prognosis, two out of four cases of ovarian PiMHEC showed advanced stage and resulted in death of disease within six months and eight weeks. This is consistent with the exceedingly poor prognosis observed in endometrial PiMHEC. The other two cases had no available follow-up data. The poor prognosis of PiMHEC suggests the importance to recognize this entity in the common practice. In our case, the tumor showed extraovarian extension but was not as advanced as the previously published cases. Moreover, the lack of follow-up data (our case is recent) prevent to draw conclusions about the biological behavior of our case.

Based on the described features, we believe that the high-grade solid basaloid appearance with ghost cell keratinization may represent the crucial morphological criterion to diagnose PiMHEC. The diffuse β-catenin expression and the loss of Müllerian markers expression may be adopted as necessary immunohistochemical criteria. We cannot exclude that some extent of PAX8 and hormone receptor expression is possible in PiMHEC, but we think that a diffuse positivity for these markers is very unlikely. We think that the presence of necrosis and the expression of CDX2 are also likely to be found in most cases of PiMHEC. Partial positivity for neuroendocrine markers, as well as the presence of a low-grade endometrioid component or an endometrioid-type precursor lesion, might be considered as desirable but non-necessary diagnostic features. Cytokeratin 7 and basal/squamous markers do not appear helpful in the diagnosis of PiMHEC as they were inconsistently expressed.

Regarding differential diagnosis, we identified two main mimickers of PiMHEC in our practice. The first mimicker is represented by cases of “conventional” high-grade endometrioid carcinomas showing a solid pattern and geographic necrosis. Tumor cells with basaloid-like features, as well as squamous differentiation, may also be observed in these cases. Morphologically, these tumors can be differentiated from PiMHEC based on the absence of the prominent ghost cell keratinization. Immunohistochemically, these tumors lack the diffuse nuclear β-catenin expression of PiMHEC. Furthermore, they generally show at least partially retained expression of Müllerian markers (Figure 2e,f). The second mimicker is represented by low-grade endometrioid carcinoma showing diffuse morular metaplasia with ghost cell keratinization. In fact, morular metaplasia in endometrioid carcinoma may mimic a solid growth pattern and lead to a misdiagnosis of high-grade tumor. Moreover, the presence of ghost cell keratinization is not uncommon in morular metaplasia. In these cases, immunohistochemistry should be carefully assessed, as morular metaplasia typically shows nuclear β-catenin and CDX2 expression and may be negative for Müllerian markers [15]. It should be noted that morular metaplasia is characterized by cells with bland nuclear features and wide eosinophilic cytoplasm, while the cells of PiMHEC are basaloid with high-grade features and high nucleus-to-cytoplasm ratio. Moreover, in low-grade endometrioid carcinoma there is typically an admixture of morular metaplasia with low-grade glandular elements. A low-grade glandular component can be present in PiMHEC, but only as a distinct component which do not mix with the basaloid component. Immunohistochemistry highlights such a difference: In low grade-endometrioid carcinoma, the low-grade glandular elements retain the Müllerian markers expression and lacks the nuclear expression of β-catenin and CDX2 (Figure 2g,h). In PiMHEC, there is a diffuse nuclear expression of β-catenin and CDX2 (Figure 2c,d) and a diffuse loss of Müllerian markers. Finally, geographic necrosis is usually absent in low-grade endometrioid carcinomas. These potential mimickers are not uncommon. In our literature review, we found several published cases of low-grade endometrioid carcinomas with morular metaplasia and ghost cell keratinization potentially mimicking PiMHEC [16,17,18]. Another potential mimicker is undifferentiated/dedifferentiated carcinoma, which shows a solid pattern, high-grade monomorphic cells with high nucleus-to-cytoplasm ratio, geographic necrosis, loss of Müllerian markers and may show nuclear β-catenin expression. Unlike PiMHEC, these tumors typically lack ghost cell keratinization, are composed of dyshesive cells, and show loss of epithelial differentiation (loss of e-cadherin and cytokeratin expression). In addition, two thirds of undifferentiated/dedifferentiated carcinomas show loss of SWI/SNF complex proteins SMARCA4/BRG1, SMARCB1/INI1, and ARID1B [2,7,19]. On this account, a careful evaluation of all the distinctive morphological and immunophenotypical features of PiMHEC appears necessary for a correct diagnosis.

Regarding the role of prognostic factors, several markers have been proposed to stratify prognosis in ovarian carcinoma [20,21,22]. It is worthwhile to remark that a specific molecular classification only exists for ovarian high-grade serous carcinoma [23]. As discussed above, ovarian endometrioid carcinoma might be stratified by using the four molecular groups of endometrial carcinoma [4]. Given that most PiMHEC fall into the “no specific molecular profile” group [8], further markers might be necessary to separate PiMHEC from less aggressive entities.

In conclusion, PiMHEC is an uncommon gynecological neoplasm which can arise in the endometrium and in the ovary. Despite showing evidence of derivation from endometrioid-type lesions, PiMHEC displays peculiar histological and immunohistochemical features and a highly aggressive behavior, which may warrant its recognition as a distinct entity. Defining precise diagnostic criteria is therefore crucial to identify PiMHEC and differentiate it from mimickers. In this study, we have proposed possible diagnostic criteria, but we acknowledge that larger case series are needed to evaluate these points. Further studies are strongly encouraged in this field.

## Figures and Tables

**Table 1:** Clinicopathological features of the current case and previously published cases of putative ovarian PiMHEC.

Case	Age	Tumor Diameter	Tumor Extent	Low-Grade Component	Putative Precursor	Follow-Up
Fang 1996	48	13 cm	Confined to the ovary	Absent	Endometriosis	Not reported
Lalich 2010	31	9 cm	Abdominopelvic carcinomatosis with visceral and cutaneous metastases	Present	Not reported	DOD at 6 months
Zamecnik 2014	45	14 cm	Nodal and visceral metastases	Absent	Not reported	DOD at 8 weeks
Current case	65	12 cm	Tubal involvement	Present	Seromucinous borderline tumor	Recent case

DOD: dead of disease.

**Table 2:** Immunohistochemical features of the current case and previously published cases of putative ovarian PiMHEC.

Case	Β-Catenin	CDX2	Müllerian Markers	Neuroendocrine Markers	Basal/Squamous Markers
Fang 1996	Not assessed	Not assessed	Not assessed	NSE+ (patchy) Synaptophysin+ (patchy)	Not assessed
Lalich 2010	Diffuse nuclear staining	Not assessed	ER- PR- CK7-	Chromogranin- Synaptophysin-	p63- CK5/6- CK34βE12-
Zamecnik 2014	Diffuse nuclear staining	Not assessed	ER- PR- CK7-	Chromogranin- Synaptophysin-CD56-	p63+ CK5/6+ CK34βE12+
Current case	Diffuse nuclear staining	Diffuse nuclear staining	ER- PR- PAX8-CK7+	Chromogranin+ (focal) Synaptophysin+ (patchy)	P63+ (focal)

## Data Availability

Additional data are available from the corresponding author upon reasonable request.
